# Portuguese validation of the Adult Separation Anxiety—Questionnaire (ASA-27)

**DOI:** 10.1371/journal.pone.0248149

**Published:** 2021-03-10

**Authors:** Antonio Ruiz-García, Óliver Jiménez, Davinia María Resurrección, Marco Ferreira, José Reis-Jorge, Javier Fenollar-Cortés

**Affiliations:** 1 Department of Psychology, Universidad de Córdoba, Córdoba, Spain; 2 Department of Personality, Assessment and Psychological Treatment, Faculty of Psychology, Universidad de Málaga, Málaga, Spain; 3 Department of Psychology, Universidad Loyola Andalucía, Seville, Spain; 4 Instituto Superior de Educação e Ciências, ISEC Lisboa, Lisboa, Portugal; Medical University Innsbruck, AUSTRIA

## Abstract

Adult separation anxiety disorder (ASAD) is characterized by developmentally inappropriate and excessive fear or anxiety concerning separation from those to whom the individual is attached. Despite the high rates of this diagnosis among Portuguese adults, there is a lack of measures to assess it. In this study, we assessed the psychometric properties of a Portuguese adaptation of the Adult Separation Anxiety questionnaire (ASA-27) on a sample of 267 adults (72.7% women) aged 18–80 years (*M* = 40.5, *SD* = 13.1). Factor structure, internal consistency, and convergence validity were examined. This study confirmed the single-factor structure of the Portuguese version of ASA-27. Consistency was high for the total sample (ω = .92) and by gender (ω = .93 and 92, men and women groups, respectively). The scale was positively related to the Portuguese version of State-Trait Anxiety Inventory (STAI) (*r* = .57, *p*< .001, for both State and trait anxiety scales) and Composite Codependency Scale total score (*r* = .29, *p*< .001). In addition, the ASA-27 total score showed incremental validity in the explanation of anxiety measured by STAI. In conclusion, results show that the Portuguese version of the ASA-27 is a reliable and valid measure of ASAD.

## Introduction

Separation anxiety disorder (SAD) was considered a childhood and youth disorder until the fifth edition of the Diagnostic and Statistical Manual of Mental Disorders (DSM-5) [1–3]. Nowadays, DSM includes the onset in childhood but also occurring during adulthood, with different clinical manifestations [4–8]. Additionally, a recent study found that a third of the diagnoses during childhood continued during adulthood [9].

Adult separation anxiety disorder (ASAD) showed similar clinical manifestations than in the children population. Specifically, in adults, the symptoms of the disorder are high anticipatory anxiety about symbolic or real separation from the home or attachment figures [10], offspring leaving the parental home, the end of a relationship [11] or moving to a new city. In addition, thoughts of harm or worry about befalling the attachment figures may occur, particularly in cases of separation from, or need to stay in touch with the attachment figures [12–14]. Several studies have highlighted that almost 43% of people reported the onset of a SAD during their adulthood, more specifically around their twenties [15, 16].

Different studies have found that ASAD has been associated with other anxiety and emotional disorders during adulthood [17] and a higher prevalence of panic disorder or bipolar type 2 disorder [18]. ASAD is associated with several comorbid clinical disorders such as post-traumatic stress disorder, anxiety, prolonged grief, emotional or personality disorders [13, 19–24]. Several studies have found a relationship between ASAD and panic disorder [12, 25–28]. In patients with panic disorder or generalized anxiety disorder, the comorbidity between the diagnosis and ASAD is almost 40% [29]. In this line, other studies have found that ASAD was comorbid with bipolar type 1 disorder [25, 30,31].

The lifetime prevalence of ASAD in the general population is 4.8%, being higher in low-middle income countries (5.5%) than in middle- and high-income countries (4.7%) [32]. Higher prevalence was found in Colombia and the United States (9.8% and 9.2%, respectively). In Europe prevalence rates are 1.2% in Spain, 2% in Germany and 3.5% in France. Noteworthy is the prevalence in Portugal (6.4%) with one of the highest rates in high-income countries [32]. However, these studies have a major limitation: they were conducted with retrospective cohorts and assessed ASAD employing a modification of the DSM-IV A criteria of SAD to adapt it to adults [33]. This limitation needs to be taken into account and there is a need to carry out new prospective cohort studies with the DSM-5 criteria [4].

ASAD prevalence is higher among women than men [32]. Specifically, the proportion of cases is higher among women with low-middle sociocultural status who might have overcome adversities during childhood, lived in a dysfunctional family environment or been exposed to traumatic experiences [9]. These risk factors can trigger the development of the SAD both during childhood and adulthood [9]. Specifically, in Portugal the lifetime prevalence is higher among women (7.2%) than in men (5.7%) [32].

Despite the relevance of the ASAD, there are few instruments designed to assess this disorder in the adult population. The first instrument to evaluate ASAD was the Adult Separation Anxiety Questionnaire (ASA-27) [34] based on the first clinical interview designed to assess this disorder. The ASA-27 questionnaire contains 27 items with a four Likert-type response options and has been adapted to the Turkish population [35] and Spanish population [33]. Both validations have shown adequate psychometric properties. Cronbach’s alpha internal consistency were .92 and .93 for the Spanish validation and the Turkish validation, respectively [33, 35]. The Spanish validation loaded on a single factor as the original validation of the ASA-27 (33, 34].

Despite the prevalence of ASAD, to our knowledge, there is no previous Portuguese validated questionnaire to assess this disorder. Therefore, the purpose of this study was to develop a Portuguese version of the ASA-27. Specific objectives were: (a) to adapt ASA-27 questionnaire to the Portuguese population; (b) to assess the psychometric properties, internal consistency, and convergent validity; and (c) to provide clinical criteria for the clinical population.

## Materials and methods

### Participants

The whole sample (N = 267) included 73 men (27.3%) with a mean age of 46.1 years (*SD* = 14.1) and 194 women (72.7%) with a mean age of 38.3 (*SD* = 12.0) years. The overall mean age of the combined sample was 40.5 years (*SD* = 13.1). They were mostly employed (82.4%), with a bachelor’s degree or higher (79.8%), living with their own family (59.9%), and having no treatment (74.2%) (see Table 1). The sample was large enough given the model structure with a single latent variable [36].

**Table 1:** Demographic characteristics of Portuguese sample (n = 267).

Gender	
Men	79 (27.3%)
Women	194 (72.7%)
Age (years)	
18–35	100 (37.4%)
36–50	103 (38.6%)
51–80	64 (24.0%)
Education	
Less than high school	38 (14.2%)
High school	7 (2.6%)
Some college	9 (3.4%)
Bachelor’s degree or higher	213 (79.8%)
Marital Status	
Single	104 (39.0%)
Married	109 (40.8%)
Divorced	35 (13.1%)
Widowed	2 (0.7%)
Other	17 (6.4%)
Work Status	
Employment	220 (82.4%)
Unemployment	7 (2.6%)
Student	29 (10.9%)
Retired/Pensioned	11 (4.1%)
Medical Status	
Psychological treatment	15(5.6%)
Psychiatric treatment	10(3.7%)
Other medical treatment	44(16.5%)
No treatment	198(74.2%)
Home environment	
Own family	160(59.9%)
With parents	29(22.1%)
Friends/Roommates	13(4.9%)
Alone	25(9.4%)
Other	10(3.7%)

### Measures

The instrument ASA-27 was translated to Portuguese with the parallel back-translation procedure [37, 38] independently by two bilingual researchers (MF and JRJ). First, the items were translated from English to Portuguese and discrepancies were solved among the two researchers. Second, the items were translated back into English by two trained translators and compared with the original ones. Finally, two undergraduate students were inquired about the comprehension and adequacy of the items. We found no major difficulties with the semantic equivalent of the items in the Portuguese version (see S1 Table in S1 File). In order to assess convergent validity of the questionnaire, several instruments assessing similar constructs have been used, such as trait-state anxiety, attachment and emotional dependence.

State-Trait Anxiety Inventory (STAI) [39, 40]. This inventory assesses state and trait anxiety with 40 Likert-type items. The Portuguese validation showed a good internal consistency for both State anxiety and Trait anxiety (Cronbach’s alpha .80 and .90, respectively).

Composite Codependency Scale (CCS) [41, 42]. This scale assesses codependence with 19-items Likert-type scale from 1 (strongly disagree) to 5 (strongly agree). This scale has three subscales and a total scale, all showing good internal consistency (Cronbach’s alpha: interpersonal control = .67; self-sacrifice = .74; and emotional suppression = .75; total scale = .80).

### Procedure

Data collection was done through an online survey. The online questionnaire included socio-demographic outcomes: age, gender, educational level, home environment, work status, marital status, medical status.

The inclusion criteria were being an adult (>18 years old), having Portuguese nationality, and being able to understand Portuguese. In order to include the largest possible number of participants from different age groups and backgrounds we started by sending the questionnaire to our private and professional contacts and asked them to forward it to their own contacts. From 326 responses, 59 of them were excluded for not meeting the criteria. The sample was recruited using a snowball sampling method. There was no reward for participation in the study.

Participants were informed and provided informed consent to participate in the study. The study protocol was approved by the Ethics Commission of Direção Geral para a Investigação e Desenvolvimento, ISEC Lisboa.

### Data analysis

Descriptive statistics, Exploratory Factor Analysis (EFA), Confirmatory Factor Analysis (CFA), internal consistency, and incremental validity were calculated both with the overall sample; analyses were also conducted by gender and age range groups. Given the data distribution a Principal Axis Factor was chosen as factor extraction method [43]. The scree plot was used to determine the number of factors to retain–looking for the break point in the data where the curve flattens out-, a procedure recommended by Costello & Osborne (2005) [44]. Minimum values of 0.32 for factor loadings [45] and 0.80 for KMO statistic were stablished [46]. Confirmatory factor analysis (CFA) was used to confirm the unique factor structure (all items loading on a single factor). Because of the lack of normality, the sample size (N > 200), and the ordinal data, mean and variance adjusted weighted least squares (WLSMV) was selected [47]. Fit measures were computed, such as the χ2 statistic, the root mean square error approximation (RMSEA), the CFI, and the TLI. The standard for good model fit was as follows: (a) CFI ≥ 0.95, (b) RMSEA ≤ 0.06, and (c) SRMR ≤ 0.06 [48]. Internal consistency was assessed using McDonald’s ω [49], defined as the proportion of item variance explained by the latent factors, and Cronbach’s α (to compare with the original scale reliability results). McDonald’s ω was calculated omitting each of the items, with the inclusion criterion that the internal consistency should be no greater than that of the scale without the omission of items.

Possible differences between groups (gender and age range groups, respectively) were also tested. Mann-Whitney analyses were run, and the effect size was calculated. A correlation of .10 is considered a small effect, a correlation of .30 is considered a medium effect, and a correlation of .50 is considered a large [50].

Partial correlation analyses (aged adjusted) were carried out between the total score for the Portuguese version of ASA-27 and the rest of the scales for convergent validity. Regarding incremental validity, hierarchical linear regression models were tested by steps to explore whether the Portuguese version of ASA-27 had a unique predictive power over STAI subscales total scores compared with CCS total score.

CFAs were computed using Mplus [51], and the rest of the analyses were computed with JASP version 0.11.1 [52].

## Results

### Factor analyses and reliability

Descriptive statistics for the items included in the Portuguese version of ASA-27 are shown in Table 2.

**Table 2:** Descriptive statistics of the Portuguese version of ASA-27.

Items	*M*	*SD*	*Mdn*	Skew	Kurt
Item 1	1.10	1.08	1	0.57	-0.98
Item 2	0.36	0.62	0	1.73	2.64
Item 3	0.45	0.93	0	2.02	2.74
Item 4	0.78	0.80	1	0.81	0.15
Item 5	0.62	0.77	0	1.18	0.98
Item 6	0.76	0.81	1	0.98	0.63
Item 7	0.88	0.84	1	0.80	0.17
Item 8	1.22	0.91	1	0.44	-0.55
Item 9	0.54	0.74	0	1.30	1.29
Item 10	0.40	0.76	0	2.09	3.91
Item 11	0.57	0.71	0	1.21	1.39
Item 12	0.61	0.88	0	1.37	0.96
Item 13	0.54	0.85	0	1.53	1.46
Item 14	0.87	0.84	1	0.83	0.19
Item 15	0.18	0.47	0	3.03	11.1
Item 16	1.18	0.93	1	0.53	-0.48
Item 17	0.99	0.78	1	0.71	0.51
Item 18	0.97	0.86	1	0.63	-0.19
Item 19	0.12	0.44	0	4.48	22.4
Item 20	0.18	0.53	0	3.42	12.8
Item 21	0.30	0.64	0	2.45	6.26
Item 22	0.62	0.78	0	1.31	1.53
Item 23	0.75	0.89	1	1.06	0.34
Item 24	0.22	0.52	0	2.69	7.93
Item 25	0.63	0.81	0	1.26	1.10
Item 26	0.42	0.70	0	1.75	2.91
Item 27	0.34	0.69	0	2.18	4.33

*Note*. ASA-27 = Adult Separation Anxiety Questionnaire; *M* = mean; *SD* = Standard Deviation; *Mdn* = Median; *Skew* = Skewness; *Kurt* = Kurtosis. Items scores ranging from 0 (*this has never happened*) to 3 (*this happens very often*).

The factors tested (variance, covariance, mean, median, standard deviation, skewness, kurtosis and Standardized Factor Loadings) are presented in Table 3.

**Table 3:** Variance (diagonal), covariance and standardized factor loadings of items from CFA of the Portuguese version of the ASA-27.

Item	1	2	3	4	5	6	7	8	9	10	11	12	13	14	15	16	17	18	19	20	21	22	23	24	25	26	27	*Factor Loading*
1	1.17																											.518
2	.12	.38																										.501
3	.15	.09	.86																									.431
4	.19	.12	.15	.64																								.516
5	.23	.09	.15	.21	.59																							.677
6	.21	.09	.20	.26	.27	.66																						.674
7	.25	.17	.09	.31	.21	.24	.71																					.635
8	.35	.15	.20	.15	.23	.27	.29	.83																				.750
9	.16	.09	.12	.16	.17	.15	.23	.25	.54																			.549
10	.21	.09	.12	.15	.23	.13	.19	.29	.16	.57																		.608
11	.14	.09	.17	.15	.16	.30	.19	.27	.13	.20	.51																	.662
12	.40	.12	.08	.18	.31	.29	.23	.24	.14	.17	.17	.78																.621
13	.24	.07	.01	.11	.18	.13	.19	.17	.15	.11	.11	.30	.72															.487
14	.34	.17	.24	.26	.30	.33	.36	.44	.26	.20	.26	.32	.26	.71														.856
15	.15	.09	.09	.10	.16	.12	.13	.17	.11	.11	.10	.14	.10	.18	.22													.784
16	.27	.07	.19	.21	.28	.41	.24	.38	.14	.17	.30	.29	.23	.39	.15	.86												.672
17	.18	.13	.14	.19	.15	.24	.31	.35	.21	.14	.26	.19	.12	.37	.12	.29	.60											.694
18	.34	.12	.20	.22	.26	.27	.28	.44	.20	.29	.26	.24	.18	.43	.14	.35	.34	.73										.774
19	.09	.05	.04	.08	.06	.06	.08	.07	.05	.04	.04	.14	.11	.09	.06	.07	.05	.08	.20									.501
20	.13	.05	.03	.06	.13	.10	.14	.13	.08	.12	.06	.20	.17	.17	.10	.16	.10	.13	.10	.28								.671
21	.22	.10	.09	.16	.22	.20	.18	.21	.15	.13	.16	.20	.15	.27	.14	.24	.17	.20	.06	.15	.41							.813
22	.19	.11	.14	.17	.24	.29	.22	.27	.16	.16	.22	.21	.18	.35	.12	.32	.27	.26	.07	.14	.25	.61						.700
23	.26	.13	.19	.10	.24	.19	.25	.43	.20	.30	.25	.18	.18	.37	.11	.30	.24	.43	.03	.09	.21	.26	.79					.704
24	.13	.06	.11	.09	.17	.15	.13	.16	.16	.11	.14	.16	.09	.20	.13	.16	.14	.16	.06	.09	.24	.19	.15	.27				.827
25	.25	.13	.16	.19	.21	.24	.22	.38	.24	.18	.22	.20	.14	.37	.13	.31	.31	.37	.08	.10	.21	.29	.32	.21	.65			.739
26	.19	.05	.05	.06	.19	.13	.14	.14	.04	.20	.13	.13	.12	.19	.08	.18	.13	.14	.02	.13	.16	.17	.14	.12	.07	.49		.444
27	.10	.06	.10	.03	.11	.10	.12	.28	.13	.23	.14	.12	.07	.20	.08	.16	.15	.26	.03	.08	.10	.16	.35	.08	.18	.10	.47	.609

**Fig 1:**
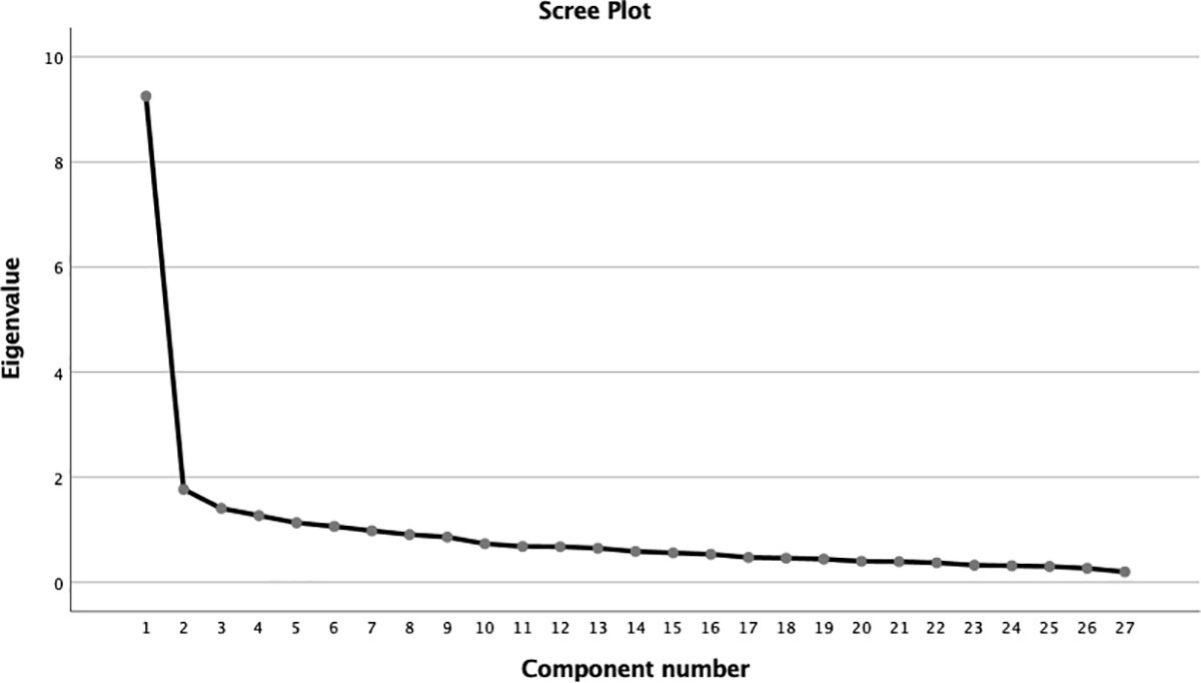
Scree plot for the Exploratory Factor Analysis (EFA) of the Portuguese validation of the Adult Separation Anxiety—questionnaire (ASA-27).

EFA suggested a single-factor structure with items loading from .322 (tem 3) to .798 (item 14). As can be seen in Fig 1, the curve quickly flattens out after the first factor. The explained variance for the first factor was 32.5% (a possible second factor explained 4.7% of the variance). The goodness of fit of the EFA was adequate (KMO = .915).

The unidimensional model was proposed for the CFA (every item loading on a single factor), the same as in the original validation of the measure [34]. The unique factor model showed adequate fit indices for the overall sample and all subsamples (see Table 4). For the overall sample, CFI and TLI were higher than .90—the minimum value in order to ensure that misspecified models are not accepted [48]—but slightly lower than .95. RMSEA value was lower than .06. Relative normed chi-square was within the adequate range (χ2/df = 1.91). Item residual variances ranged between .186 (item 3) and .733 (item 14); therefore, there were no negative values for residual variances. As evidence of reliability, the omega coefficient was high (ω = .92); that is, more than 90% of item variance was explained by the latent factor.

**Table 4:** Goodness-of-fit and reliability indices for the Portuguese version of ASA-27 obtained by gender and age-range groups.

	*df*	χ^2^	CFI	TLI	RMSEA [95%CI]	ω
Overall sample	324	617.6	.941	.936	.058 [.051-.065]	.92
By gender						
Men	324	484.4	.918	.912	.082 [.067-.097]	.93
Women	324	533.0	.944	.939	.058 [.049-.066]	.92
By age ranges						
18–35	324	467.2	.935	.930	.066 [.053-.079]	.92
36–50	324	384.7	.971	.969	.043 [.022-.058]	.93
51–80	324	443.0	.896	.888	.076 [.057-.093]	.92

*Note*. ASA-27 = Adult Separation Anxiety Questionnaire; χ^2^ = Chi-square fi statistic; CFI = comparative fit index; TLI = Tucker-Lewis Index; RMSEA = root mean square error approximation; ω = McDonald’s omega.

When the CFAs were re-run by groups, mixed results were found. However, it should be considered that samples were quite small for some groups. So, we should be careful in the interpretation of these results. While the larger sample groups (i.e., «women», «18–35» and «36–25» groups) showed adequate goodness-of-fit, the shorter sample groups (i.e. «men» and «51–80» groups; both groups with fewer than 100 cases) showed poor goodness-of-fit.

### Differences between age and gender

Kolmogorov-Smirnov tests indicated that ASA-27 score did not follow a normal distribution (D(267) = .130, *p*< .001), so non-parametric statistics were calculated. The analysis revealed the existence of differences in ASA-27 scores according to gender (*U* = 5845.0, *p* = .028) but not for the age range group (χ^2^(2) = 5.20, *p* = .074). The Mann-Whitney test indicated that ASA-27 scores were higher for women (*Mdn* = 14.5) compared with men (*Mdn* = 13.0), but the effect size was small (*r* = -.16). Regarding the post-hoc analysis for age range, there were significant differences between «18 to 35» (*Mdn* = 15.5) and «36 to 50» groups (*Mdn* = 13.0) in ASA-27 median scores (*U* = 6066.5, *p* = .028) with small effect size (*r* = .18). There were no differences between the «51 to 80» group and the rest of the age range groups.

### Convergent and incremental validity correlations

Age was significantly correlated with ASA-27 the Portuguese Version of ASA-27 (*r* = -.13, *p* = .032), STAI Trait (*r* = -.13, *p* = .044), and Self-Sacrifice (*r* = .17, *p* = .007). No more significant correlations were found. Spearman and Partial correlations (age-adjusted) between the Portuguese version of the ASA-27 with STAI and CCS scales are displayed in Table 5. All correlations between ASA-27 and STAI scales were large and significant. Results were similar by gender. Higher scores on both the state and trait anxiety were associated with significantly higher scores on the Portuguese version of the ASA-27 for every sample (overall, men and women samples). Correlations were slightly higher in the women sample than in the other samples.

**Table 5:** Convergent validity of the Portuguese version of the ASA-27 (age adjusted).

	*Overall sample*	Men	Women
STAI			
State anxiety	.57***	.44***	.60***
Trait anxiety	.57***	.45***	.59***
CCS			
Interpersonal Control	.29***	.19	.33***
Self-Sacrifice	.20**	.07	.23**
Suppression of Emotions	.19**	-.00	.27***
Total score	.29***	.11	.36***

*Note*. ASA-27 = Adult Separation Anxiety Questionnaire; STAI = State Trait Anxiety Inventory (Portuguese version); CCS = Composite Codependency Scale (Portuguese version)

**p < .01

***p < .001

However, while all the correlations between ASA-27 and CCS total score and subscales were significant for the overall sample (small to medium effect), results were different by gender samples. Both for overall and women samples, higher scores on ASA-27 were significantly associated with higher scores on CCS total score and subscales. In contrast, scores on the CCS total and subscales were not significantly related to ASA-27 scores for the men sample.

Regarding incremental validity, Table 6 shows the results from the hierarchical linear regression models to explore the unique predictive value of the Portuguese version of ASA-27 over CCS on STAI. Gender, but not age, was a substantive predictor of STAI scales. When CCS subscales were added, Suppression of Emotions subscale was a better predictor of STAI subscales than the gender (although gender improved their standardized regression coefficients). In the next step, ASA-27 score was added, and the magnitude of the standardized regression coefficients for the Suppression of Emotions subscale was reduced, although still significant. The ASA-27 added moderate incremental improvements to the amount of variance accounted by the models on scores of STAI scales (ΔR2 = 23, p < .001 for STAI-State; ΔR2 = 24, p < .001 for STAI-Trait). Therefore, the Portuguese Version of the ASA-27 had a moderate to high unique predictive value, relative to the CCS on anxiety both as trait and state.

**Table 6:** Hierarchical regression analyses on anxiety measures.

Outcome	Step	Predictor	β	*R*^2^	Δ*R*^2^
STAI-State	Step 1	Gender	.12	.03	-
		Age	-.09		
	Step 2	Gender	.17**		
		Age	-.11		
		CCS	.32***	.13	.12***
	Step 3	Gender	.08		
		Age	-.04		
		CCS	.16*		
		ASA-27	.51***	.36	.23***
STAI-Trait	Step 1	Gender	.13*		
		Age	-.09	.03	-
	Step 2	Gender	.17**		
		Age	-.11		
		CCS	.32***	.13	.10***
	Step 3	Gender	.08		
		Age	-.05		
		CCS	.17**		
		ASA-27	.51***	.37	.24***

*Note*. ASA-27 = The Self-Report Questionnaire for Adult; STAI = State Trait Anxiety Inventory (Portuguese version); CCS = Composite Codependency Scale (Portuguese version) Total score.

*p < .05

**p < .01

***p < .001

## Discussion

In the last two decades, the relevance of separation anxiety in adulthood has been highlighted [6]. In this context, the creation and development of new instruments adapted for the adult population is needed.

The main objective of the present study was to develop and to analyse the psychometric properties of a Portuguese version of the ASA-27 with a non-clinical sample of Portuguese adults. This is the first instrument available to assess ASAD in the Portuguese population. Overall, the psychometric properties of the instrument were good when considering a one factor structure. The analysis of the factor structure yielded conclusive results, as in the other validation studies and the original instrument [33–35].

The factorial structure of the questionnaire has been shown to be unifactorial, that is, all items load in a single factor, as in the original version [34] and in its adaptations to Turkish [35] and Spanish [33]. However, in this study the adjustment was slightly higher in the RMSE index for men, although confirmatory factor analyses by groups (men and women) have been performed in small groups. Therefore, it would be necessary to confirm it with larger samples of both men and women. However, the adjustment is adequate for the total sample.

Our results support the instrument’s convergent validity, with a total ASA-27 score correlated positively with co-dependency scale and both anxiety state and trait. The data yields interesting results regarding gender and age. ASA-27, in both the overall sample and women sample, correlated positively with CCS total and subscales scores, but these results were not significant for the men group. However, ASA-27 correlated positively with the STAI both in trait and state also in the men sample. Yet these results were not found in previous adaptations [33, 35], which might support the fact that ASAD is associated with being women [20]. These differences might show different profiles between genders. Despite group differences, the model showed an appropriate goodness-of-fit.

Due to the absence of specific instruments to assess ASAD among the Portuguese population, the present study has important theoretical implications. This study yields empirical support to the notion of the new diagnosis of ASAD recently included in DSM-5. In addition, the availability of this scale has practical implications allowing a more accurate diagnosis of anxiety problems comorbid with ASAD. The availability of this instrument can be very useful for clinicians in evaluating other cases, seeing their psychological intervention improved when considering emotional aspects such as separation anxiety, which are usually not considered when addressing other psychological problems such as other types of anxiety problems, or depressive problems [53]. In this regard a recent study found that ASAD was highly prevalent in non-responders to standard anxiety treatment [49]. The development of instruments to assess this novel diagnosis can provide novel information about separation anxiety being a transdiagnostic variable. Therefore, properly detecting separation anxiety problems must be an important objective to improve treatments and their effectiveness, to solve clinical issues.

Some limitations should be considered. Firstly, despite the sample size, a snowball procedure was followed reflecting some consequences of a convenience sample. Data collection could be improved through using for example random sampling procedures. Secondly, although above the minimum for the implemented psychometric techniques, it would be useful to test psychometric properties with larger men samples or with different educational levels to increase the power of the analyses. Finally, it would be interesting for future studies to examine the relationship of ASA-27 with other related constructs such as dependent personality. Future studies should address normative data of the ASA-27 Portuguese for clinical population.

## Conclusion

The main scientific benefit of using this scale validation analysis is developing the habit of asking fundamental questions about the construct investigated. In this study, ASA-27 Portuguese was found to be a reliable and valid instrument in assessing the presence of separation anxiety symptoms in adults. The time spent to complete the scale is reduced, which can increase its reliability. This instrument can be useful to obtain accurate information about the diagnosis of SAD in adulthood. In other words, the results can be consistently reproduced. Furthermore, the satisfactory internal consistency and stability scores supported its good reliability. All the findings support the conclusion that ASA-27 questionnaire is an adequate measure of ASAD and can be used with confidence among the Portuguese population.

## Supporting information

S1 File (DOCX)
