# Supplementary material for: Portuguese validation of the Adult Separation Anxiety—Questionnaire (ASA-27)
Source: PLoS One. 2021 Mar 10;16(3):e0248149. doi: 10.1371/journal.pone.0248149 (PMC7946201; doi:10.1371/journal.pone.0248149)
Supplement: S1 File — (DOCX) [file pone.0248149.s001.docx]

**S1 Table.**

**Portuguese version of the ASA-27**

Os estados que se seguem referem-se a características emocionais que poderá ter experimentado na idade adulta (após os 18 anos de idade). Para cada questão, escolha o correspondente. Por favor, responda a todas as perguntas.

|  |  | Acontece com muita frequência | Acontece com alguma frequência | Acontece ocasionalmente | Nunca acontece |
| --- | --- | --- | --- | --- | --- |
| 1. | Tem-se sentido mais seguro(a) em casa quando está acompanhado(a) com pessoas que lhe são próximas? |  |  |  |  |
| 2. | Tem sentido dificuldade em ficar afastado(a) de casa durante algumas horas seguidas? |  |  |  |  |
| 3. | Tem transportado na sua carteira ou bolsa algo que lhe dá uma sensação de segurança ou conforto? |  |  |  |  |
| 4. | Tem-se sentido demasiado stressado(a) antes de sair de casa para uma longa viagem? |  |  |  |  |
| 5. | Tem sofrido de pesadelos ou sonhado com a separação de alguém próximo de si? |  |  |  |  |
| 6. | Tem-se sentido demasiado stressado(a) quando alguém que lhe é próximo vai viajar? |  |  |  |  |
| 7. | Tem ficado perturbado(a) quando a sua rotina diária habitual é alterada? |  |  |  |  |
| 8. | Tem-se preocupado com a intensidade do seu relacionamento com as pessoas mais próximas de si, por exemplo pessoas com quem tem uma forte ligação? |  |  |  |  |
| 9. | Tem tido sintomas como dores de cabeça, dores de estômago ou náuseas (ou outros) antes de sair para trabalhar ou para outra actividade regular fora de casa? |  |  |  |  |
| 10. | Acha que fala demasiado só para manter as pessoas junto de si? |  |  |  |  |
| 11. | Tem-se sentido especialmente preocupado(a) em saber para onde vão as pessoas que lhe são próximas, por exemplo quando as deixa para ir trabalhar ou para qualquer outro sítio? |  |  |  |  |
| 12. | Tem tido dificuldade em dormir à noite quando está sozinho(a) em casa, ex. o seu sono é melhor se alguém próximo de si está em casa? |  |  |  |  |
| 13 | Tem notado que é capaz de adormecer melhor se ouvir as vozes das pessoas com quem vive ou o som da TV ou do rádio? |  |  |  |  |
| 14. | Tem ficado muito angustiado(a) quando pensa em estar afastado(a) de pessoas próximas de si? |  |  |  |  |
| 15. | Tem sofrido de pesadelos ou sonhos com estar longe de casa? |  |  |  |  |
| 16. | Tem-se preocupado muito com o que possa acontecer de mau às pessoas que lhe são próximas, por exemplo ter um acidenmte de carro ou sofrer de uma doença fatal? |  |  |  |  |
| 17. | Tem ficado aborrecido(a) quando alterações na sua rotina diária interferem no contacto com as pessoas que lhe são próximas? |  |  |  |  |
| 18. | Tem-se preocupado muito com a possibilidade de as pessoas de quem gosta se afastarem de si? |  |  |  |  |
| 19. | Tem percebido que dorme melhor com as luzes de casa ou do quarto acesas? |  |  |  |  |
| 20. | Tem evitado ficar em casa sozinho(a) especialmente quando as pessoas que lhe são próximas se encontram ausentes? |  |  |  |  |
| 21. | Tem sofrido ataques súbitos de ansiedade ou de pânico (por exemplo, tremores, suores, falta de ar, palpitações) quando pensa deixar as pessoas que lhe são próximas ou essas pessoas deixarem-no(a) a si? |  |  |  |  |
| 22. | Tem percebido que fica ansioso(a) quando não fala ao telefone regularmente, por exemplo diariamente, com as pessoas que lhe são próximas |  |  |  |  |
| 23. | Tem receado não ser capaz de lidar ou seguir em frente se uma pessoa de quem gosta o/a deixar? |  |  |  |  |
| 24. | Tem sofrido ataques súbitos de ansiedade ou de pânico (por exemplo, tremores, suores, falta de ar, palpitações) quando está separado de pessoas que lhe são próximas? |  |  |  |  |
| 25. | Tem-se preocupado demasiado com possíveis situações que obriguem a separação de pessoas que lhe são próximas, por exemplo exigências de trabalho? |  |  |  |  |
| 26. | As pessoas que lhe são próximas fazem-lhe notar que "fala muito"? |  |  |  |  |
| 27. | Tem-se preocupado com o facto de a sua relação com algumas pessoas ser tão próxima que lhes possa causar problemas? |  |  |  |  |

**S2 Table.**

**English version of the ASA-27[1]**

|  |  | This happens very oftern | This happens fairly often | This happens occasionally | This has never happened |
| --- | --- | --- | --- | --- | --- |
| 1. | Have you felt more secure at home when you are with people that are close to you? |  |  |  |  |
| 2. | Have you experienced difficulty in staying away from home for several hours at a time? |  |  |  |  |
| 3. | Have you been carrying around something in your purse or wallet that gives you a sense of security or comfort? |  |  |  |  |
| 4. | Have you experienced extreme stress before leaving home to go on a long trip? |  |  |  |  |
| 5. | Have you suffered from nightmares or dreams about being separated from someone close to you? |  |  |  |  |
| 6. | Have you experienced extreme stress before leaving someone close to you when going away on a trip? |  |  |  |  |
| 7. | Have you become very upset when your usual daily routine is disrupted? |  |  |  |  |
| 8. | Have you been worried about the intensity of your relationship with  those people closest to you, eg. that you are too strongly attached? |  |  |  |  |
| 9. | Have you experienced symptoms such as headaches, stomach-aches or nausea (or other) before leaving for work or other regular activity outside the home? |  |  |  |  |
| 10. | Do you find that you talk a lot in order to keep people close to you? |  |  |  |  |
| 11. | Have you been especially concerned about where people close to you are going when you are separated from them, eg. when you leave them to go to work or go out of the house? |  |  |  |  |
| 12. | Have you experienced difficulty in sleeping alone at night, eg. is your  sleep better if someone close to you is in the house? |  |  |  |  |
| 13 | Have you noticed that you are better able to go off to sleep if you can hear the voices of people you are close to or the sound of the TV or the radio? |  |  |  |  |
| 14. | Have you become very distressed when thinking about being away from people that are close to you? |  |  |  |  |
| 15. | Have you suffered from nightmares or dreams about being away from home? |  |  |  |  |
| 16. | Have you been worrying a lot about people close to you coming to serious harm, for example, meeting with a car accident, or suffering from a fatal illness? |  |  |  |  |
| 17. | Have you become very upset with changes to your usual daily routine if they interfere with your contact with persons close to you? |  |  |  |  |
| 18. | Have you been worrying a lot about people you care about leaving you? |  |  |  |  |
| 19. | Have you found that you sleep better if the lights are on in the house or in the bedroom? |  |  |  |  |
| 20. | Have you tried to avoid being at home alone especially when people close to you are out? |  |  |  |  |
| 21. | Have you suffered from sudden bouts of anxiety or panic attacks (eg. sudden shaking, sweating, shortness of breath, pounding heart) when thinking about leaving people close to you or about them leaving you? |  |  |  |  |
| 22. | Have you found that you get anxious if you do not speak to people that are close to you on the telephone regularly, eg. daily? |  |  |  |  |
| 23. | Have you been afraid that you would not be able to cope or could not go on if someone you cared about left you? |  |  |  |  |
| 24. | Have you suffered from sudden bouts of anxiety or panic attacks (eg. sudden shaking, sweating, shortness or breath, pounding heart) when separated from people close to you? |  |  |  |  |
| 25. | Have you been worrying a lot about possible events that may separate you from those close to you eg. because of work requirements? |  |  |  |  |
| 26. | Have people close to you mentioned that you ‘talk a lot’? |  |  |  |  |
| 27. | Have you been worrying that your relationships with some people are so close that it may cause them problems? |  |  |  |  |

# References

1. Manicavasagar V, Silove D, Wagner R, Drobny J. Adult Separation Anxiety Questionnaire - ASA-27. Measurement Instrument Database for the Social Science. 2012. Retrieved from www.midss.ie
